# Supporting a student with visual impairment in the intensive care unit

**DOI:** 10.4102/sajp.v75i1.1324

**Published:** 2019-07-31

**Authors:** Michael Rowe, Tania Steyl, Joliana Phillips, Anthea Rhoda

**Affiliations:** 1 Department of Physiotherapy, Faculty of Community and Health Sciences, University of the Western Cape, Cape Town, South Africa; 2 Faculty of Community and Health Sciences, University of the Western Cape, Cape Town, South Africa

**Keywords:** visual impairment, intensive care unit, physiotherapy education, disability, intensive care

## Abstract

**Background:**

The Department of Physiotherapy at the University of the Western Cape began accepting students with visual impairments (VIs) into the undergraduate physiotherapy programme in 1996. However, until recently, none had received a clinical rotation in any high care setting.

**Objectives:**

The aim of this study was to explore the experiences of all stakeholders involved in the process of placing a student with VI into the intensive care unit (ICU).

**Method:**

This case study used interviews with relevant stakeholders to explore their experiences of integrating the ICU placement into the student’s clinical programme. Interviews were recorded and transcribed, and then analysed thematically.

**Results:**

There was a certain amount of anxiety present, especially among clinical staff, before the placement began. Discussions among stakeholders at each stage of the process served to identify potential problems before they arose, and allowed staff to plan solutions in advance. Challenges were found in both the attitudes of staff, and in the clinical environment, some of which were relatively easy to address, while others will require significant investments of resources to resolve.

**Conclusion:**

Our findings suggest that it may be possible to successfully place students with VI into intensive care settings, and they can enjoy positive learning experiences, given an appropriate context and adequate support. However, care needs to be taken at every stage of the process to ensure that supporting structures are in place prior to, and during, the placement.

**Clinical implications:**

All stakeholders, including the students and the relevant clinical and academic staff, need to be actively involved in the process of planning for the clinical placement.

## Introduction

In 2008, South Africa ratified the United Nations Convention on the Rights of Persons with Disabilities (UNCRPD), thus committing the government to facilitate education and employment for citizens with impairments that affect their participation in society at all levels. The UNCRPD laid the foundation for the more recent White Paper on the Rights of Persons with Disabilities (2015), which aims to remove discriminatory barriers to access and participation for persons with disabilities in South Africa. These commitments apply not only to those seeking equitable employment but also to those seeking access to higher education opportunities. However, students with disabilities still only form a small percentage of the total number of higher education enrolments (Matshedisho 2007). While some progress has been made in mainstreaming people with disabilities (PWDs) into policies and programmes over the past 20 years, these initiatives are not always sustainable as they rely more on individual action than planned and coordinated interventions (White Paper 2015).

In addition to the lack of systemic implementation of mainstreaming, there may also be reluctance from professional departments in higher education institutions (HEIs), who feel that they do not have the skills, experience or access to resources to accommodate students with visual impairments (VIs). These include trained peer mentors, the availability of textbooks or course materials in electronic formats and the ongoing training of academic staff with respect to alternative teaching practices. This is an important consideration because these factors are essential to students’ success (Frank, McLinden & Douglas 2014; Johnston et al. 2016).

The process of mainstreaming calls for a redesign of all aspects of the curriculum to ensure that a wide range of people, including those with various categories of disability, are able to participate without the need for individual adaptations to be made (White Paper 2015). There is therefore a pedagogical implication for integrating students with disability into the undergraduate curriculum, in that the changes must be made throughout the programme, which will have more far-reaching implications for educators.

There are legitimate challenges for departments who wish to place students with VI into acute care settings, such as the intensive care unit (ICU). These concerns usually centre around aspects of practice that are traditionally thought to require ‘normal’ vision, and may include reading the output of monitors, medical records and patient charts. In addition, there are concerns that students with VI may not be well placed to respond appropriately in situations that require an ability to notice small details in the patient’s rapidly changing clinical context (Johnston et al. 2016). Clinicians and educators may be apprehensive to have students working within the acute care setting because of the fact that it is a fast-paced and dynamic environment, which requires students and clinicians to respond accordingly.

However, even though there are legitimate concerns by educators and clinicians with respect to students’ performance in acute care settings, there is also evidence that some countries have been able to successfully integrate students with VI into these settings. Most notably, this progress has been made in the UK, where there is a long history of educating physiotherapists with VI, initially in a segregated setting (Thomas 1957) but more recently as part of the mainstream undergraduate programme (Atkinson & Hutchinson 2013). However, student success in this challenging environment is premised on the provision of extensive support, as well as positive attitudes from peers and clinical staff (Atkinson & Hutchinson 2013; Johnston et al., 2016). This support should be in the form of addressing physical, social and attitudinal issues that may serve as barriers to student success (World Confederation for Physical Therapy 2016).

The Department of Physiotherapy at the University of the Western Cape (UWC) has been at the forefront of this movement in South African physiotherapy education, accepting and supporting students with VIs since 1996. However, even though students with VI were accepted into the programme, they had never been placed in the ICU as part of their clinical rotations. In 2014, the Health Professions Council of South Africa (HPCSA) made it a requirement that all undergraduate physiotherapy students must manage critically ill patients who required ventilatory support (HPCSA 2014). This requirement, along with the White Paper (2015), places South African physiotherapy departments in a position where they must actively seek to enrol students with VI into the undergraduate programme, and also accept the additional challenges of supporting these students in acute care settings. In 2015, the Department of Physiotherapy at UWC placed a student with VI into a high care setting as part of a clinical rotation, which we believe represents the first time in South Africa that this has been described. The aim of this article is to describe the experiences of all stakeholders involved in the process of placing the student in the ICU.

## Method

To align the department with the government’s position on non-discrimination against students with VI, as well as the HPCSA requirement that all undergraduate students receive training in high care clinical contexts, the department began planning for all students – regardless of visual acuity – to have a clinical rotation in the ICU during 2015. The VI coordinator at UWC, clinician on the clinical ICU placement, clinical supervisor and student prepared for the placement with meetings and clinical visits to begin negotiating the expectations of all stakeholders (Hutchinson & Atkinson 2010; Steyl 2010). The clinical coordinator in the university department met with the head of the hospital physiotherapy department prior to the placement to discuss any concerns with respect to patient safety and student support.

In addition, the student and clinical supervisor visited the ICU before the start of the rotation so the student could familiarise himself with the environment and to interact with a patient in an informal session. In this way, the time required to read the patient’s folder, conduct an assessment, provide treatment and document the findings was determined, which influenced the planning for the placement. The student would also be able to identify possible barriers that emerged from his perspective during the visit. Blank bed charts were given to the student to take home so that he could familiarise himself with the format. It should be noted that this student had partial vision and was able to read notes and bed charts independently with magnification. Finally, the student was provided with additional support in the form of a full-time supervisor who was present for the full duration of the time that he spent in the ICU. This was different for most other students, who spend only a few hours a week with their supervisors.

This study used a case study design with in-depth interviews of all the relevant stakeholders to ensure that a broad and comprehensive set of experiences was captured. This study took place in a variety of locations but interviews were conducted primarily in the physiotherapy department on campus. The student’s clinical rotation was in a medical ICU at a tertiary hospital in the Western Cape, which included rotations into a ward for patients with neurological impairment. The sample of participants in this study included the single undergraduate student (S1), one supervising clinician from the clinical placement (CS1), the UWC clinical coordinator and student academic supervisor (CC1) and the VI coordinator in the department (VI1). The interviews were conducted by a colleague who was not employed by the university or clinical placement, using a self-developed interview guide that was informed by the literature (Johnston et al. 2016).

The data were analysed using thematic analysis (Braun & Clarke 2006) where the transcribed data were read and analysed by two of the authors to determine the initial codes. These codes were collated into potential themes that were independently reviewed by a third author.

### Ethical considerations

This study was approved by the University of the Western Cape Research Ethics Committee (project registration number: 15/6/80).

## Results

The content analysis identified various themes as they related to the experiences of the stakeholders that emerged from the transcribed data. These included *stakeholder attitudes, situational challenges* and *environmental challenges*. Quotes are presented in support of the themes, and include both positive and negative points of view.

### Stakeholder attitudes

It was evident that the student had a positive attitude towards the ICU clinical placement, as well as towards his impairment demonstrated by his proactive position and initiative in terms of wanting to work in the ICU.

‘So you know that the student approached me and asked me why is he not getting a clinical rotation in ICU? So it was his initiative to come to us.’ (CS1, female, 35 years old)

Despite his positive attitude, it was clear that the student found the idea of a clinical rotation through an ICU block intimidating, not only because of his VI but also because of the nature of the placement itself, which most students find challenging.

‘Initially I think all students have this stigma of ICU as difficult.’ (S1, male, 26 years old)

The student was also fully aware of the obstacles that he might encounter because of his VI:

‘And at first, yes, it is difficult because your attention must be on everything at once, and I think with me, I was particularly a bit scared because due to my visual impairment I was not sure if I would be able to have my attention at the patient, at the monitors, making sure I don’t pull out lines by accident, and all that stuff.’ (S1, male, 26 years old)

Attitudes of staff at the clinical facility were mixed. Some clinical staff at the hospital were reluctant to allow a student with a VI onto the ICU, reported by the clinical coordinator after their meeting:

‘I spoke to [*a senior staff member*] and he was very negative…from [*his*] side – it was: this person is disabled and this person should not be allowed in the ICU.’ (CC1, female, 45 years old)

Despite the reluctance of some staff, a workable solution was found by combining the ICU placement with another clinical rotation so that the student would not spend all of his time in the ICU:

‘So he was at a neuro placement and would go into the ICU twice a week, once with me and once with the clinician – and the reason being again with the shortage of staff and all the students being in ICU in the morning it was felt that because he needed special attention it would be easier to take him in at 12h00 or 13h00 when everybody else had left the ICU, it was quieter and the clinician had more time.’ (CC1, female, 45 years old)

The student’s ability to work independently was also questioned and of great concern in a resource-constrained health system.

‘… he wasn’t able to work independently; he needed constant supervision because of that [*the visual impairment*].’ (CS1, female, 35 years old)

Even though the student developed the ability to perform certain tasks independently, this did not change the clinician’s view about constant supervision being needed by the student, as required by the nature of the patients being treated in the ICU. Although it should be noted that this would generally be true of many students, regardless of their visual acuity.

‘So some of the things he was able to do with time …, he became less dependent as such … but because of the concern in ICU and the critical nature of the place and the patients, it was felt that supervision was required …’ (CS1, female, 35 years old)

But the clinician also understood that this was a trial and realised the potential of the student to be successful in the ICU, as described by the clinical coordinator:

‘I think where the more positive feedback came was the fact that he had come for that trial and I think the clinician could see that there was potential in the student and that is why it was allowed in the end.’ (CC1, female, 45 years old)

In addition to the physiotherapy clinicians, the student perceived that the nursing staff were helpful:

‘First of all the staff was very helpful. Wherever I needed assistance they were more than glad to help me.’ (S1, male, 26 years old)

This sentiment was echoed by the clinical coordinator when she stated that:

‘[*T*]he nurses went out of their way to help him. [*One member of staff*] would [*indistinct*], there’s the monitor, and the portable monitor – I think that was nice.’ (CC1, female, 45 years old)

Support was also offered by those not specifically involved in the process of supervising the student. Patients in the setting also expressed their admiration and support for the process, as described by the clinical supervisor:

‘The feedback that I got from the patients was, wow, this is fantastic, this is a great step that you’re taking. We admire your university for doing this, for supporting the student and trying to see if we can place visually impaired students in ICU.’ (CS1, Female, 35 years old)

### Environmental factors

Environmental factors that were highlighted by the participants included concerns around the student’s ability to make observations and the physical placement of equipment in the room. There were also concerns regarding the potential danger to patients as there are areas of practice in acute care environments where impairment in vision may put the student at a disadvantage:

‘I think in ICU it’s very tricky to the point where it can potentially be dangerous … You [*the student*] might not notice as quickly if something’s happening … it takes much longer to notice something’s wrong.’ (VI1, male, 30 years old)

The above sentiment was echoed by the supervising clinician as some of the patients were intubated and therefore would not have been able to communicate verbally with the student, thus forcing the student to rely on his vision for different situational indicators:

‘So a lot of our cues that we take are what we see, whether it’s expression, lip-reading … it’s something we take for granted; you don’t realise how much of it actually plays a role.’ (CS1, female, 35 years old)

The interaction between the monitor and bed placement, time of day (and associated position of the sun) and the student’s photophobia resulted in a challenge that was dependent on the environmental variables:

‘Another challenge was where the patient was situated in the room in relation to where the windows were. The blinds could not adjust … Also the glare on the screen made it difficult to actually read it [*the monitor*].’ (S1, male, 26 years old)

The clinician also noted that the fixed monitors were challenging for the student:

‘Some of the monitors could not be lowered, so it presented an extra problem.’ (CS1, female, 35 years old)

The student echoed the notion that reading the monitors posed a significant challenge for him, not only because of the fixed position, but also because of the contrast in colour:

‘In the ICU obviously the monitor was set up quite high and it’s fixed, so it can’t move down, which made it really difficult to actually read everything.’ (S1, male, 26 years old)

However, there were some positive points that demonstrated how small changes in the environment led to improvements in the student’s’ performance. For example, staff members could enlarge the font size on some of the monitors:

‘On some of them [*the monitors*] we could change the size of the numbers … some of them, because of the colour he [*the student*] couldn’t see it regardless that it was a very bright colour.’ (CS1, female, 35 years old)

‘But although on the monitor that is mounted, we can enlarge the readings, it’s still difficult, I think more due to the contrasts in colours; … I had a difficult time to read the invasive blood pressure which was red on black.’ (S1, male, 26 years old)

Attempts were made to adjust the environment by providing the student with a mobile monitor although there were additional challenges that need to be considered if this were to be used more consistently:

‘I had to connect the portable monitor myself…there were no parameters set for alarms, so the alarm would beep the whole time.’ (S1, male, 26 years old)

## Discussion

One of the most striking aspects of this project was the initiative taken by the student to drive the process.

His positive attitude towards his VI was one of the factors that may explain his taking the initiative and approaching the department and asking why he was not considered for an ICU placement. His enthusiasm for practice and the profession meant that he was not satisfied with the practices and discourses in which disability relationships are held. He was comfortable challenging the status quo in the department (Campbell 2011). In this respect, the attitude and personal confidence of the student may have been an important factor in his successful rotation in the ICU.

However, rather than suggesting that student placement in high or intensive care settings should only be considered based on students’ personal attributes, physiotherapy departments should instead work with students from the time that they enter into the programme in order to prepare them for clinical work in high care settings (Atkinson & Hutchinson 2013; Hutchinson & Atkinson 2010). This could be in the form of getting input from the student on the details of their VI, as well as their perceptions of working in a critical care environment (Atkinson & Hutchinson 2013). In other words, it may not be reasonable to mandate that this rotation be required for all students and decisions should be made on a case-by-case basis.

Having said that it is also worth considering that students, particularly those with severe impairments, should be given the opportunity to discuss any concerns they may have with an ICU clinical rotation (Hutchinson & Atkinson 2010). In other words, it may not be reasonable to insist that all students with VI participate in an ICU rotation, although this will depend on the particular rules and policy of professional bodies and the local context. The student in this study was positive and enthusiastic to the extent that he initiated this process. However, it is also important to acknowledge that not all students with VI will be comfortable working in the ICU, so it is especially important for departments to work with students prior to, and during, the placement. The HPCSA (2014) requirement that all students must experience the management of critically ill patients on ventilatory support may complicate the matter, because it seems potentially to conflict with the rights of students with disabilities to access professional programmes.

Supporting a student with VI in the acute care setting requires more than simply providing physical access to the environment and epistemological access to the necessary information. The attitudinal barriers from staff may do more to limit students’ success than any environmental challenge. In this study, there was evidence that some staff members in the ICU had a clear bias against the placement of the student in that setting. This is not a problem that is limited to South African physiotherapy practice. In the UK, where there is a long history of clinical placement for students with VI, there is evidence that they regularly encounter prejudice and discrimination (Atkinson & Hutchinson 2013). It may be that employers’ and colleagues’ fear, anxiety and lack of awareness about disability may also erect barriers to full participation of employees with disabilities (ibid.). In other words, it is not only the physical aspects of access that can prevent successful integration of students and clinicians with VI into the ICU. There is therefore a clear need to do more in order to challenge the negative stereotypes associated with students with VI who are placed in the ICU setting.

From this study, it was very clear that regular communication during the preparation phase was an essential component of the project. This communication was instrumental in ensuring the mutual understanding that was necessary for successful outcomes, especially between the clinical coordinator and the supervising clinician at the placement, as misunderstanding may have led to confusion and under preparedness of all stakeholders. It is very important to note that the clinical staff and environment must also be prepared in advance and that there needs to be an understanding that it cannot be business as usual in the ICU (Hutchinson & Atkinson 2013). In this study, the support provided to the student included adapting the physical environment, how the clinical rotation was structured, and the way that the student interacted with patients. Thus, preparedness is not only the responsibility of the student and academic institution but it rather implies that all relevant stakeholders be included in the preparation.

In this study, even though the student demonstrated competence to practise independently after working for a period of time, clinicians in the placement still required him to have constant supervision ‘because of that’ (i.e. because of his VI). While other students may have been given leave to practise without supervision for some periods of time, the basis for the decision around constant supervision for this student seems to have been his VI. We suggest that if the student has demonstrated competence in the clinical setting, then care should be taken to ensure that he or she is treated in the same way as any other student. It is important that educators and clinicians have the same expectations of all students. There is evidence that many students will find the initial period of their clinical placements to be challenging and clinicians and clinical supervisors should not assume that a disability will necessarily make a student less proficient or more clumsy (Hutchinson & Atkinson 2010). For example, a student who has a VI may, for example, be much more mindful of the presence of catheters, drains and trailing wires in an ICU and take far greater care than students who are not disabled. Having said that, we acknowledge that this was a highly motivated student who received additional clinical and emotional support and that this may not be sustainable given additional students with VI.

It is not enough to simply provide access to the ICU as a learning environment if there is not an associated set of changes in policy and procedure. These changes include empowering the student to participate actively in the process, as well as ensuring that there is equality in the assessment of outcomes (White Paper 2015). It is important to differentiate between being equal, where all processes are the same, and equality, which ensures that students with disabilities are not disadvantaged or discriminated against by virtue of their disability. An example would be to ensure that the student with VI has more time to review the medical folder, which is fair but not equal with respect to other students. In addition, clinicians – and possibly patients – may need to be supportive of the need for students with VI to make use of technology to enhance their learning and practice strategies (Hutchinson & Atkinson 2010). These include portable devices for text and monitor magnification, audio recording devices to enable recording of the patient interaction and subsequent note taking. We acknowledge that there are ethical considerations that need to be taken into account for some of these options but also suggest that they are not inherently problematic, provided that all stakeholders agree on the conditions of use.

## Conclusion

This study demonstrated that the integration of students with VI into the ICU is both necessary and possible, although there are significant challenges to be overcome in the process. To align with national policies of non-discrimination, South African physiotherapy departments should not only actively campaign for the recruitment of undergraduate students with VI into the programme but also ensure that support frameworks are present during the students’ progression through the system, especially related to the emotional support that may be necessary during stressful periods such as in the intensive care setting. This support should not only be in the form of providing physical and epistemological access to the clinical platform but also in working against the embedded stereotypes and biases that often inform decision-making around issues of student clinical placement. Students who are to be placed in ICU and other high care settings should be actively involved in the process from the beginning and should be invited to spend time in the ward prior to the beginning of the rotation. Clinicians, clinical supervisors and educators all need to be aware of the additional support and time that students with VI require, and acknowledge that it may not be sustainable over the long term and with increasing numbers of students with VI in the cohort.

Admitting students with VI into professional programmes like physiotherapy involves elements of participation, accountability, non-discrimination, empowerment and an express linkage to human rights standards. There seems to be, if not a legal requirement then at least a moral one, to offer students with such a disability the opportunity to participate in their training at every level. However, if physiotherapy departments are going to take the decision to integrate students with VI into ICU and high care clinical settings, there is an associated responsibility to ensure that appropriate support is provided, which has logistical, pedagogical and financial implications.
